# Predictive Factors of Biliary Tract Cancer in Anomalous Union of the Pancreaticobiliary Duct

**DOI:** 10.1097/MD.0000000000003526

**Published:** 2016-05-20

**Authors:** Jin-Seok Park, Tae Jun Song, Tae Young Park, Dongwook Oh, Hyun Kyo Lee, Do Hyun Park, Sang Soo Lee, Dong Wan Seo, Sung Koo Lee, Myung-Hwan Kim

**Affiliations:** From the Digestive Disease Center (J-SP), Department of Internal Medicine, Inha University School of Medicine, Incheon; Division of Gastroenterology (TJS, TYP, DO, DHP, SSL, DWS, SKL, M-HK), Asan Medical Center, University of Ulsan College of Medicine, Seoul; and Department of Internal Medicine (HKL), Ilsan Paik Hospital, Inje University College of Medicine, Goyang, Republic of Korea.

## Abstract

The assessment of malignancies associated with anomalous union of the pancreaticobiliary duct (AUPBD) is essential for the design of appropriate treatment strategies. The aim of the present study is to measure the incidence of AUPBD-related pancreaticobiliary malignancy and to identify predictive factors. This retrospective cohort study included cases of 229 patients with AUPBD between January 1999 and December 2013. The impact of bile duct dilatation on the incidence of AUPBD-related pancreaticobiliary disease was measured, and predictive factors were evaluated.

Among 229 patients with AUPBD, 152 had common bile duct dilatation (≥10 mm) (dilated group) and 77 did not (<10 mm) (nondilated group). Intrahepatic cholangiocarcinoma occurred more frequently in the nondilated group than in the dilated group (3.9% vs 0%; *P* < 0.05). By contrast, no significant difference in the incidence of extrahepatic cholangiocarcinoma was observed between the 2 groups (1.3% vs 3.9%; *P* = 0.271). By univariate analysis, age, type of AUPBD, and the level of pancreatic enzymes refluxed in the bile duct were associated with occurrence of biliary tract cancers. In multivariate analysis, age ≥45 years (odds ratio [OR] 1.042, 95% confidence interval [CI] 1.011–1.073, *P* < 0.05), P-C type (OR 3.327, 95% CI 1.031–10.740, *P* < 0.05), and a high level of biliary lipase (OR 4.132, 95% CI 1.420–12.021, *P* < 0.05) showed a significant association with AUPBD-related biliary tract cancer.

Intrahepatic cholangiocarcinoma may occur more frequently in AUPBD patients without bile duct dilatation. Age ≥45 years, P-C type, and biliary lipase level ≥45,000 IU/L are significantly associated with AUPBD-related biliary tract cancer.

## INTRODUCTION

Anomalous union of the pancreaticobiliary duct (AUPBD) is a rare congenital anomaly in which the pancreatic and biliary ducts join anatomically outside of the duodenal wall, usually forming a markedly long common channel.^1^ The most clinically important feature of AUPBD is its association with carcinoma of the biliary tract, that is, bile duct and gallbladder cancer.^2^ In the literature, the incidence of bile duct and gallbladder cancer is 3.6% to 4.9% and 6.8% to 14.8% in AUPBD patients, respectively.^3–5^ In general, the stasis and regurgitation of pancreatic juice into the dilated bile duct and gallbladder is believed to be the main mechanism of carcinogenesis in AUPBD.^6^ Since the action of the sphincter muscle does not functionally affect the junction in AUPBD, persistent reflux of pancreatic juice into the biliary tract occurs. This results in recurrent inflammation of the bile duct and gallbladder epithelium, and leads to hyperplasia and metaplasia, which might induce malignant transformation of the biliary epithelium.^7^ To support this hypothesis, studies were conducted to clarify the correlation between refluxed pancreatic juice enzymes and occurrence of biliary tract cancer by measuring pancreatic enzyme levels in bile; however, these studies failed to identify a clear association between amylase levels and the development of biliary tract cancers.^8,9^

Although controversy remains regarding the mechanism of carcinogenesis, many studies demonstrated that biliary tract cancers develop preferentially at sites where there is stasis of activated pancreatic enzymes, such as in the dilated bile duct or gallbladder.^10^ In this regard, prophylactic flow-diversion surgery consisting of extrahepatic bile duct resection along with cholecystectomy is widely accepted as the treatment of choice for AUPBD patients with bile duct dilatation to prevent development of both bile duct and gallbladder cancer.^11^ For AUPBD without bile duct dilatation, prophylactic cholecystectomy alone is generally performed and advocated in many institutions owing to the concept that the stasis of pancreatic enzymes occurs mainly in the gallbladder^5^; however, some experts suggest excision of the extrahepatic biliary tract and cholecystectomy in AUPBD patients without biliary dilatation because reflux of pancreatic enzyme would still occur after cholecystectomy, thereby predisposing the patient to other biliary tract cancers.^3,12^ Therefore, the optimal surgical approach to AUPBD without bile duct dilatation remains debatable, and the occurrence of biliary tract cancer in AUPBD without bile duct dilatation needs to be clarified.

In this context, we assessed the incidence of biliary tract cancer in AUPBD patients with and without bile duct dilatation. In addition, factors predictive of biliary tract cancer in patients with AUPBD were evaluated.

## METHODS

### Patients

We conducted this study in patients with AUPBD diagnosed by endoscopic retrograde cholangiopancreatography (ERCP) from January 1999 to December 2013 at the Asan Medical Center. The patients enrolled were divided into 2 groups based on bile duct dilation. The medical records of the patients were reviewed retrospectively, and the following information was extracted: clinical characteristics, clinical course, occurrence of biliary tract cancer, ERCP findings, and level of refluxed pancreatic enzymes in the common bile duct. This study was approved by the institutional review board at Asan Medical Center.

### Definitions

**FIGURE 1:**
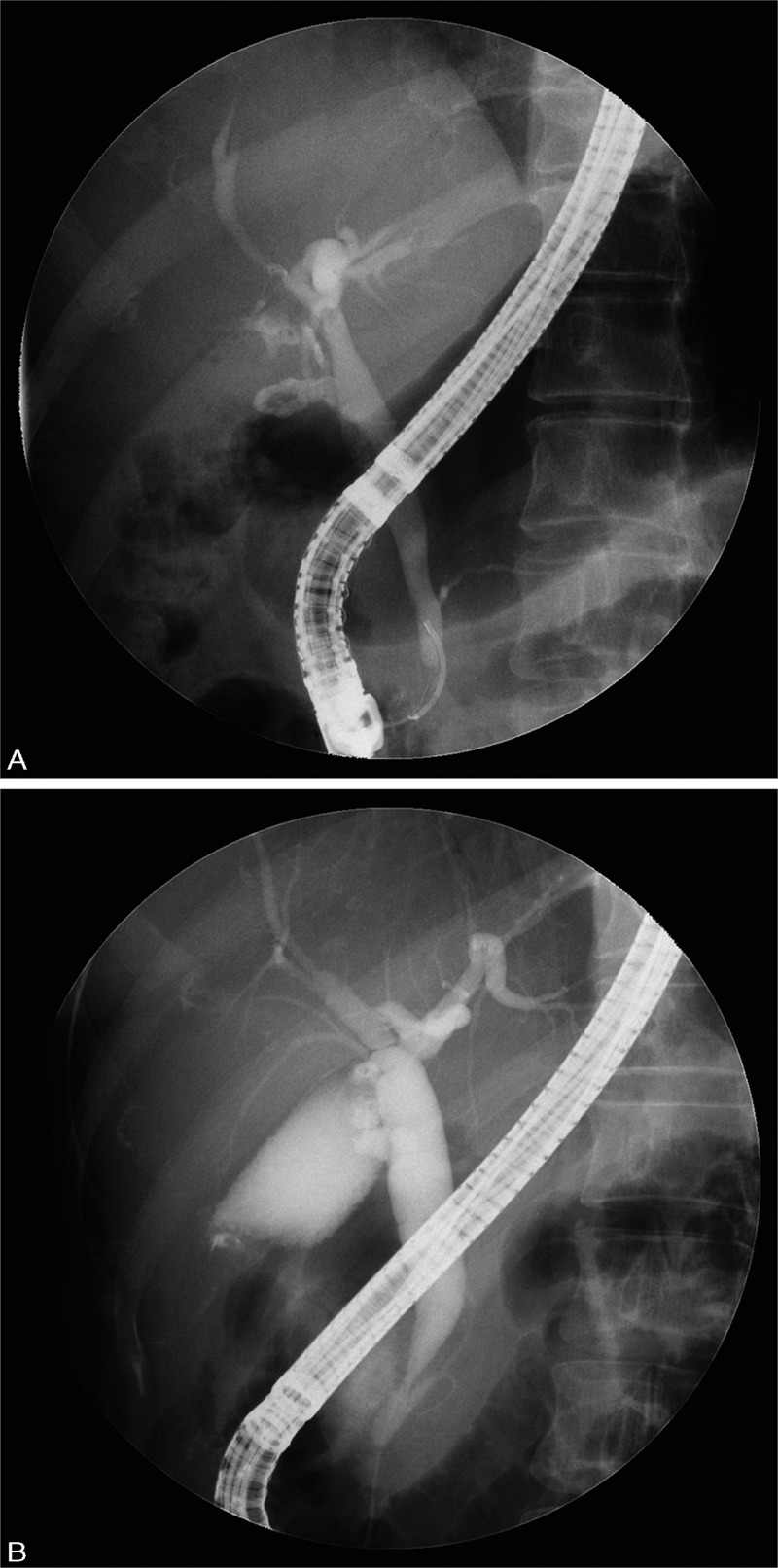
A, Endoscopic retrograde cholangiopancreatography (ERCP) shows the main pancreatic duct joins the common bile duct (P-C type). B, ERCP shows the common bile duct joins the main pancreatic duct (C-P type).

Anomalous union of the pancreaticobiliary duct was defined as a common channel greater than 15 mm in length on a cholangiogram. Bile duct dilatation was defined as an extrahepatic duct with a maximum diameter of ≥10 mm on a cholangiogram; however, if bile duct dilatation was deemed to be due to obstruction by a tumor and/or stone, it was not regarded as bile duct dilatation. AUPBD-related biliary tract cancer consists of gallbladder cancer, extrahepatic cholangiocarcinoma (ECC), and intrahepatic cholangiocarcinoma (ICC). ECC was defined as cancer arising from the hepatic duct bifurcation to the distal common bile duct. Intrahepatic biliary tract cancer was defined as cancer occurring in the bile ducts within the liver.^13^ Cases of AUPBD were classified into 2 categories: P-C type, in which the pancreatic duct joins the bile duct; or C-P type, in which the bile duct joins the pancreatic duct^1^ (Figure 1). Bile sampling was performed during ERCP at the common bile duct. The levels of amylase and lipase were measured, and the correlation between these levels and biliary tract cancers was investigated.

### Statistical Analysis

Statistical analysis was performed using SPSS (SPSS for windows, version 21.0, SPSS Inc., Chicago, IL). The baseline characteristics were assessed using the independent *t* test for continuous variables, whereas the chi-square test was used for categorical variables. Multivariate logistic regression was used to determine whether the factors affected the incidence of biliary cancer. The odd ratios (ORs) and 95% confidence intervals (95% CIs) were determined. Receiver-operating characteristic (ROC) curve analysis was used to determine cut-off values, based on highest sensitivity and specificity, for classification of patients. Due to the wide range of enzyme levels, values obtained by applying the common logarithm to amylase levels were used to calculate the OR. The appropriate lipase level cut-off value was defined by the ROC curve. The cut-off value was used to determine the relative risk associated with age and the biliary lipase level. The chi-square test was used to compare the incidence of biliary tract cancer between the groups generated by classification based on cut-off values.

## RESULTS

### Patient Characteristics

From January 1999 to December 2013, 229 patients (0.5%) were diagnosed as having AUPBD out of 46,049 ERCP referrals. The mean age of the subjects was 48.79 ± 14.08 years, and the male-to-female ratio was 2.47:1 (163:66 cases). The mean extrahepatic bile duct diameter was 17.77 ± 13.03 mm. One hundred sixty-eight (73.4%) patients had P-C type, and 61 (26.6%) had C-P type. The mean level of amylase and lipase was 89805.1 ± 187930.5 IU/L and 248231.7 ± 686426.8 IU/L, respectively. In 229 AUPBD patients, 76 patients were diagnosed with gallbladder cancer, 7 with ECC, and 3 with ICC. During the study period, a total of 1111 patients were newly diagnosed gallbladder cancer, 10,065 patients with ECC, and 3659 patients with ICC in our center. The incidence of AUPBD in gallbladder cancer, ECC, and ICC were 6.84%, 0.08%, and 0.07%, respectively.

### Clinical Baseline

Of the 229 patients, bile duct dilatation was present in 152 patients (dilated group) and absent in the remaining 77 patients (nondilated group). The mean diameter of the bile duct was 23.9 ± 14.4 mm in the dilated group and 8.1 ± 1.9 mm in the nondilated group (*P* < 0.001). No significant statistical difference in baseline clinical characteristics, including age and sex, was observed between the 2 groups. The P-C type was more common in the nondilated group than in the dilated group (*P* = 0.009). No significant differences in levels of refluxed pancreatic enzymes were observed between the 2 groups. ICC and pancreatitis were more frequent in the nondilated group. In particular, ICC was seen only in the nondilated group (*P* = 0.014). For other pancreaticobiliary diseases, including gallbladder cancer (*P* = 0.184), ECC (*P* = 0.271), and pancreatic cancer (*P* = 0.688), the incidence did not differ statistically between the 2 groups (Table 1).

**TABLE 1:**
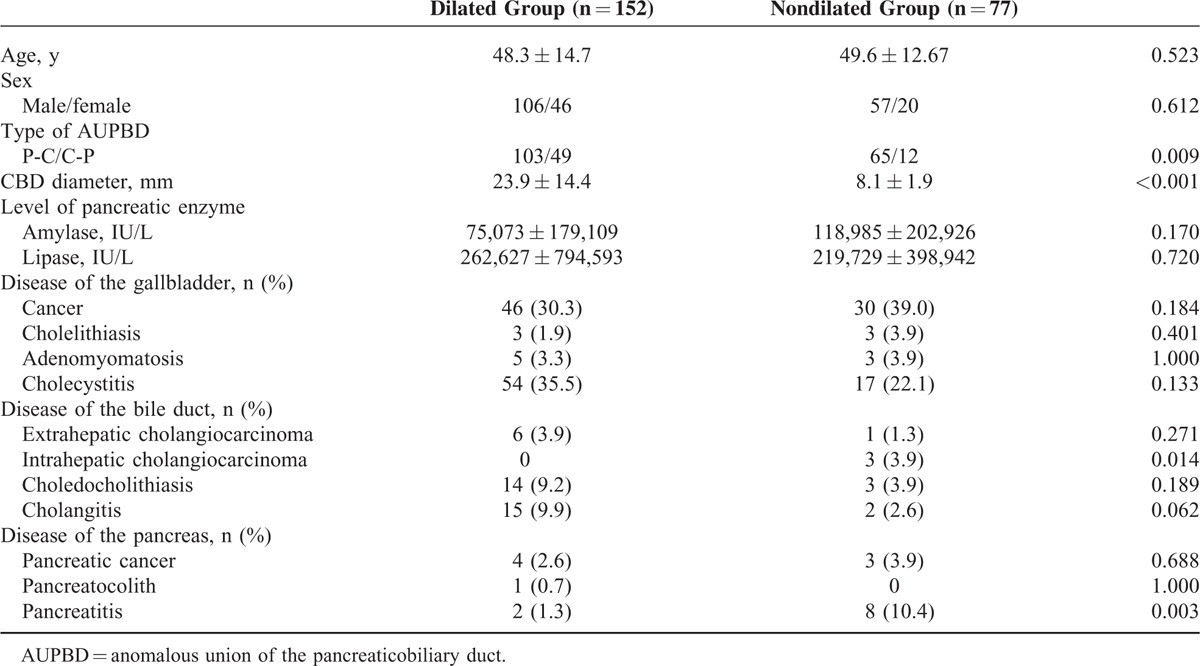
Comparison Between AUPBD Patients With and Without Bile Duct Dilation

### Factors Predictive of AUPBD-related Biliary Tract Cancer

Univariate analysis showed significant differences in age, biliary amylase level, biliary lipase level, and type of AUPBD between patients with biliary tract cancer and those without (Table 2). By multivariate analysis, age (odds ratio [OR] 1.042, 95% confidence interval [CI] 1.011–1.073, *P* < 0.05), biliary lipase level (OR 4.132, 95% CI 1.420–12.021, *P* < 0.05), and type of AUPBD (OR 3.327, 95% CI 1.031–10.740, *P* < 0.05) showed a correlation with the incidence of AUPBD-related biliary tract cancer (Table 3).

**TABLE 2:**
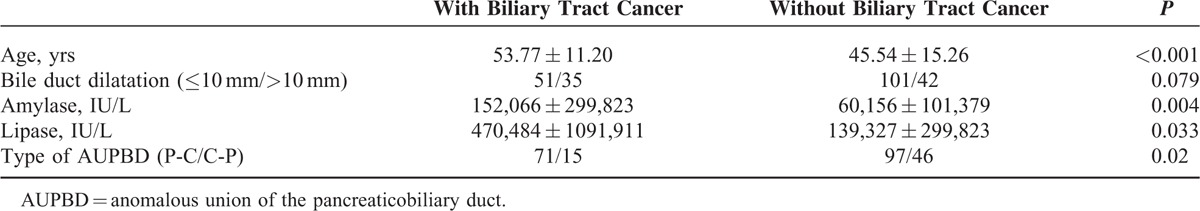
Univariate Analysis of Factors Predictive of AUPBD-related Bile Duct Cancer

**TABLE 3:**
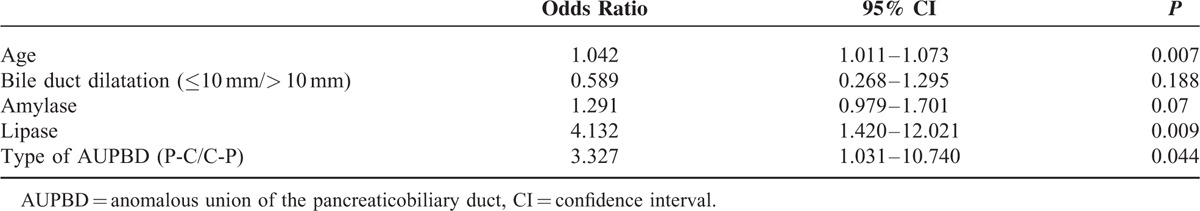
Multivariate Analysis of Factors Predictive of AUPBD-related Bile Duct Cancer

### Age and AUPBD-related Biliary Tract Cancer

**FIGURE 2:**
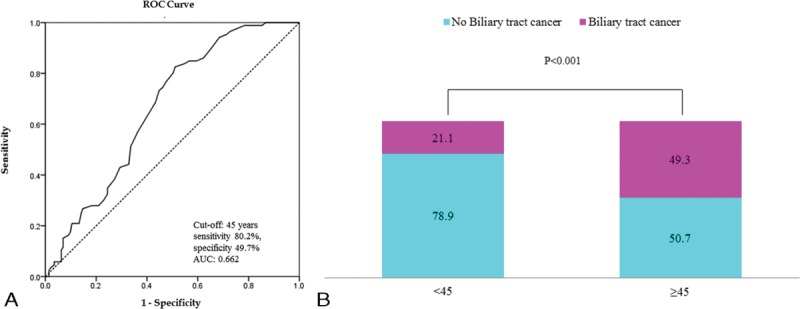
A, Incidence of biliary cancer based on patient age. B, The incidence of biliary cancer is higher in patients ≥45 years of age than in younger patients (OR 3.640, 95% CI 2.001–6.621, *P* < 0.001). CI = confidence interval, OR = odds ratio.

The mean age of AUPBD patients with biliary tact cancer (53.8 ± 11.2 yrs) was significantly higher than that of those without biliary tract cancer (45.5 ± 15.3 yrs) (*P* < 0.05). The youngest patient with biliary tract cancer was a 29-year-old woman with gallbladder cancer. By logistic regression, the odds of biliary tract cancer increased by 1.042 with each year of age (95% CI 1.011–1.073, *P* < 0.05). The age cut-off for biliary tract cancer was calculated using the ROC curve. When the best cut-off point was set at 45 years, the area under the ROC curve (AUC) was 0.662. The sensitivity and specificity were 80.2% and 49.7%, respectively. The occurrence of biliary tract cancer in patients aged ≥45 years was significantly higher than that in patients aged <45 years (OR 3.640, 95% CI 2.001–6.621, *P* < 0.05) (Figure 2).

### AUPBD Type and AUPBD-related Biliary Tract Cancer

Univariate analysis showed that biliary tract cancers were significantly associated with certain AUPBD types: the P-C type was more frequently detected in patients with biliary tract cancer (*P* < 0.05). Multivariate analysis showed that the odd of biliary tract cancer was 3.327 higher in the P-C type than in the C-P type (95% CI 1.031–10.740, *P* < 0.05). Ninety-one of 229 patients underwent surgical bile duct resection, in which 35 and 56 patients were C-P type and P-C type, respectively. The pathologic results of resected bile ducts were compared to evaluate the difference of histological features of the bile duct dependent on AUPBD types, and there were no significant differences between C-P type and P-C type (Table 4).

**TABLE 4:**
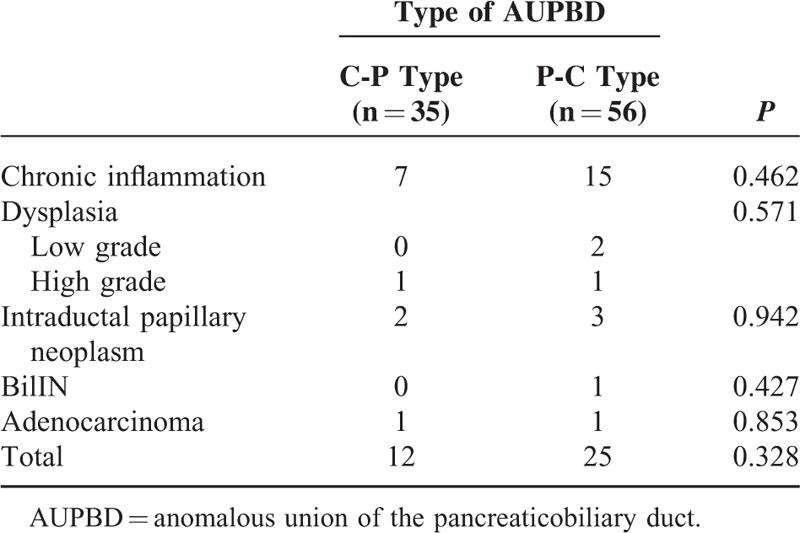
The Pathologic Findings of Bile Duct in Accordance With Types of AUPBD

### Refluxed Pancreatic Enzymes and AUPBD-related Biliary Tract Cancer

**FIGURE 3:**
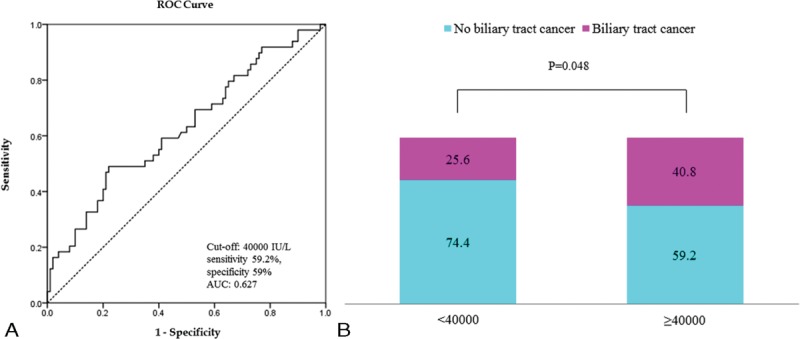
A, Incidence of biliary cancer based on biliary lipase level. B, The incidence of biliary cancer is high when biliary lipase levels are ≥40,000 IU/L (OR 2.235, 95% CI 1.085–4.604, *P* < 0.05). CI = confidence interval, OR = odds ratio.

The refluxed pancreatic enzymes were measured in 149 patients. Multiple logistic regression analysis revealed that AUPBD patients with biliary tract cancer tended to have higher lipase levels than those without biliary tract cancer (OR 4.132, 95% CI 1.420–12.021, *P* < 0.05). ROC curve analysis determined the biliary lipase level cut-off value for AUPBD-related biliary tract cancer occurrence as 40,000 IU/mL (AUC 0.627) at the highest sensitivity (59.2%) and specificity (59%) (Figure 3A). Biliary tract cancer occurred more frequently in AUPBD patients with ≥40,000 IU/mL than in those with <40,000 IU/mL (OR 2.235, 95% CI 1.085–4.604, *P* < 0.05) (Figure 3B). The amylase level did not show any significant correlation with biliary tract cancer.

### Occurrence of Bile Duct Cancers in AUPBD Patients With Predictive Factors

In this study, 41 patients had all 3 predictive factors, and among these, a bile duct cancer occurred in 23 patients. Hence, the positive predictive value for bile duct cancers in patients with all 3 predictive factors was 56.1%. In contrast, of the 146 patients who did not experience bile duct cancer, only 8 had all 3 predictable factors. However, the analysis of occurrence of each biliary tract cancer depending on predictive factors could not reach statistical significance because of the small number of each bile duct cancer (ECC, n = 7; ICC, n = 3) except for gallbladder cancer (Table 5).

**TABLE 5:**
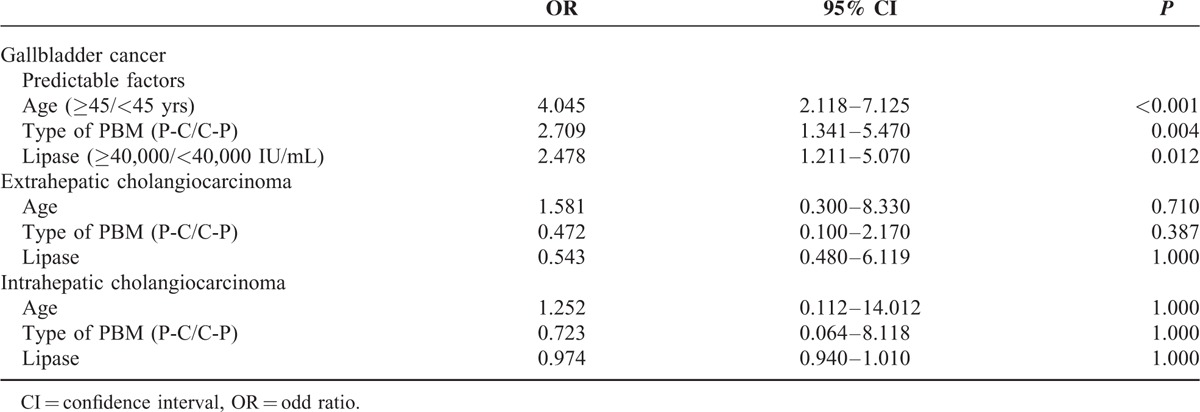
The Association Between Predictable Factors and Each Biliary Tract Cancer

## DISCUSSION

The present study focused on the incidence of biliary tract cancer in patients with AUPBD with and without bile duct dilatation, and evaluated potential predictive factors for AUPBD-related biliary tract cancer. The results showed that the risk of ICC was higher in AUPBD patients with a nondilated bile duct. This finding is of great interest and, to the best of our knowledge, is documented here for the first time. Our results also provide factors that can be used to predict AUPBD-related biliary tract cancer, including age ≥45 years, P-C type, and biliary lipase level ≥40,000 IU/L.

So far, ICC has not received significant attention in the treatment of AUPBD, because the stasis of refluxed pancreatic juice in the intrahepatic bile duct is considered to have a relatively lower risk of stasis than that in the extrahepatic bile duct or gallbladder. Although AUPBD is believed to increase the risk of ICC, limited data exist in the literature concerning the association between ICC and AUPBD. Only a few case reports have documented the occurrence of ICC in AUPBD patients.^14^ In this study, ICC occurred in 3 AUPBD patients, and all cases of ICC occurred in patients without bile duct dilatation (*P* = 0.014). This result suggests that a nondilated bile duct in AUPBD is associated with a high risk of ICC. This finding can be explained by the continuity equation, which states that the speed of a liquid is directly inversely proportional to the cross-sectional area of the vessel. Thus, because the cross-sectional area is smaller in a nondilated bile duct than in dilated bile duct, faster flow would be generated in a nondilated bile duct, resulting in refluxed pancreatic juice reaching the liver, where it may cause ICC. Therefore, we recommend that clinicians pay renewed attention to the development of ICC in AUPBD patients, particularly in those who have a nondilated bile duct.

Prophylactic bile duct resection along with cholecystectomy is the standard treatment for AUPBD with bile duct dilatation. A dilated common bile duct is considered a favorable anatomical location for the development of biliary tract cancer since stagnation of refluxed pancreatic juice is predicted to occur more frequently in a dilated duct than in a nondilated duct.^12^ The results of the present study confirm this conjecture because 6 of 7 patients with extrahepatic biliary tract cancer were in the dilated duct group. By contrast, there has been controversy over the optimal treatment for AUPBD without extrahepatic bile duct dilatation. The reason for this controversy is that the risk of developing biliary tract cancer is lower than that of developing gallbladder cancer^3,15^; however, in our study, the incidence of extrahepatic biliary tract cancer did not differ significantly between the 2 groups (*P* = 0.271), and ICC was significantly more common in patients without extrahepatic bile duct dilatation (*P* = 0.014). As a result, cholecystectomy alone may not suffice, and further surgical management including bile duct excision may be necessary. Some investigators recommended removal of the extrahepatic bile duct along with the gallbladder to prevent cancer development in the remnant nondilated bile duct.^3,16^ According to a study by Kim et al,^10^ of 55 patients with AUPBD, conducted to evaluate the occurrence of biliary tract cancer, ECC occurred more frequently in patients without bile duct dilatation than in those with bile duct dilatation (30.0% vs 4.0%; *P* = 0.015). They recommended extrahepatic bile duct excision, biloenteric anastomosis, and cholecystectomy as a treatment of choice for AUPBD without bile duct dilatation; however, routine excision of the extrahepatic bile duct in AUPBD patients presents the risk of postoperative adverse events in 9.7% to 20% of the cases.^17,18^ In the literature, extrahepatic bile duct excision is associated with increased risk of biliary anastomotic leak, biliary strictures, intrahepatic bile duct calculi, and attendant cholangitis, which are recognized as important factors affecting postoperative quality of life.^17,19,20^ Thus, factors predictive of malignancy are required to identify patients with the greatest risk of cancer in whom the risk of postoperative adverse events is outweighed by the drastic reduction in cancer risk offered by surgery.

In the present study, we found that the incidence of biliary tract cancer increases with age in patients with AUPBD. In particular, the incidence of biliary tract cancer was significantly increased in patients ≥45 years of age. The incidence of biliary tract cancer was 21% in patients between 19 and 44 years of age, and 48% in patients ≥45 years of age. A few reports on age and incidence of AUPBD-related biliary tract cancer recommended early surgical treatment for AUPBD. Kobayashi et al,^21^ who conducted research to clarify the preferable operative age in AUPBD patients with bile duct dilatation, recommended cholecystectomy or bile duct resection before the age of 40 because the youngest patient with gallbladder cancer was a 41-year-old woman. A previous nationwide survey reported that the incidence of gallbladder cancer tended to show a marked increase in AUPBD patients ≥40 years of age.^2^ Our finding has a unique advantage in that we used statistical methods to determine a cut-off age that can be used to make treatment decisions, which was not performed in other studies. In the present study, the youngest patient with biliary tract cancer was a 29-year-old woman with gallbladder cancer. On the basis of this result, the optimal age for surgery would seem to be before 29 years; however, encouraging young patients in whom biliary tract cancer has not occurred to undergo surgical treatment is very difficult. When deciding to offer surgical treatment, we should also consider morbidity, patient satisfaction, and postoperative quality of life. Therefore, the ability to determine an age cut-off for recommending prophylactic treatment is very important in clinical practice, especially for the prevention of biliary tract cancer.

In the present study, biliary tract cancer occurred significantly more often in the P-C type than in the C-P type (42.2% vs 24.6%; *P* = 0.02). A similar tendency was seen in a previous meta-analysis, which reported that 70.0% to 85.7% of gallbladder cancer patients with AUPBD belonged to the P-C type.^22^ This may be related to the direction of flow of the pancreatic juice. Since the angle between the pancreatic duct and the bile duct forms a shaft in the P-C type, the pancreatic juice may be constantly under high pressure, resulting in a continuous reciprocal reflux of pancreatic juice. By contrast, the C-P type has a right angle between the pancreatic duct and the bile duct, and the bile duct is joined to the pancreatic duct. Therefore, reflux of pancreatic juice would occur to a lesser extent than in the P-C type.

The presence of extremely high levels of pancreatic enzymes in bile could be used as a supplementary diagnostic tool to detect or predict AUPBD-related biliary tract cancer^5^; however, whether or not the level of refluxed pancreatic enzymes is predictive of a risk of developing biliary tract cancer remains questionable. According to a nationwide survey in Japan, high biliary amylase levels in AUPBD patients are not associated with biliary cancer.^23^ By contrast, Beltran et al,^24^ who conducted a study of 108 patients with normal pancreaticobiliary junction to investigate occult pancreaticobiliary reflux in benign and malignant gallbladder diseases, reported significantly higher biliary lipase levels in gallbladder cancer patients than in patients with a benign gallbladder pathology. Our results showed that the occurrence of biliary tract cancer tended to increase as the levels of pancreatic enzymes increased, and that the biliary lipase level was significantly associated with the occurrence of biliary tract cancer (OR 4.132, 95% CI 1.420–12.021, *P* < 0.05). AUPBD-related biliary tract cancer was detected more frequently in patients whose lipase levels in the common bile duct were ≥40,000 IU/L than in other patients; however, amylase levels did not show any statistical significance by multivariate analysis (OR 1.291, 95% CI 0.979–1.701, *P* = 0.07). On the basis of our results, we suggest that AUPBD patients with high levels of biliary lipase need close follow-up so as not to miss the occurrence of AUPBD-related biliary cancer.

The present study has several limitations. Firstly, it is inherently limited by its retrospective nature. Secondly, the study was conducted at a single medical center, and, thus, our results may not be generalizable. Thirdly, AUPBD was diagnosed only by ERCP, and patients who may have been diagnosed by other imaging modalities, such as magnetic resonance cholangiopancreatography, endoscopic ultrasonography, or by anatomical examination after surgery, were not included. This factor could have introduced selection bias in our results.

In conclusion, our study demonstrates that the incidence of ICC is higher in AUPBD patients without bile duct dilatation. Age, type of AUPBD, and biliary lipase level are significantly associated with AUPBD-related biliary tract cancers. Hence, clinicians should exercise caution concerning the possibility of biliary tract cancer in AUPBD patients, particularly in patients ≥45 years of age, with the P-C type and a biliary lipase level ≥40,000 IU/L. In addition, cholecystectomy and extrahepatic bile duct excision might be considered as a primary treatment for AUPBD patients with those predictive factors, even when they do not have bile duct dilatation.
